# Arrangements for the Meeting at Fort Worth, April 22, 1890

**Published:** 1890-04

**Authors:** W. A. Adams

**Affiliations:** Chair. Com. Transportation; Fort Worth, Texas


### ARRANGEMENTS FOR THE MEETING APRIL 22.

Fort Worth, Texas, April 5, 1890.

*Dr. F. E. Daniel, Editor Daniel's Texas Medical Journal:*

Dear Doctor: Please publish for the profession, that the committee on transportation have arranged with all railroads in the State, to give one and one-third fare, to the delegates and physicians who will attend the meeting in April. Very respectfully,

W. A. Adams, M. D.,
Chair. Com. Transportation.

[Tickets will be on sale at all the railroad stations; applicants for membership must bring their diploma.—Ed.]

